# Comparative study of the bronchodilator efficacy and adverse effects of salbutamol and hyoscine butylbromide in horses with severe asthma

**DOI:** 10.1111/jvim.17057

**Published:** 2024-04-12

**Authors:** Berta Mozo Vives, Sophie Mainguy‐Seers, Jean‐Pierre Lavoie

**Affiliations:** ^1^ Faculty of Veterinary Medicine, Department of Clinical Sciences University of Montreal St‐Hyacinthe Quebec Canada

**Keywords:** aerosol treatment, bronchodilator, heaves, oscillometry

## Abstract

**Background:**

Salbutamol and hyoscine butylbromide (HBB) are commonly used bronchodilators in horses with severe asthma (SA).

**Objective:**

To compare the bronchodilation potency, duration, and adverse effects of salbutamol and HBB in SA.

**Animals:**

Six horses in exacerbation of SA.

**Methods:**

The effects of inhaled salbutamol (1000 μg) and HBB (150 mg, IV) were compared in a randomized, blinded, crossover experiment. Lung function, intestinal borborygmi and heart rate were assessed before and sequentially until 180 minutes after drug administration, and analyzed with 2‐way repeated‐measures ANOVA and Dunnett's multiple comparison tests.

**Results:**

Both treatments caused a similar improvement in lung function. Pulmonary resistance and reactance returned to baseline values within 30 minutes after HBB administration, whereas salbutamol improved reactance until 180 minutes (mean improvement at 180 minutes of 0.040 Kpa/L/s, 95% CI = 0.004 to 0.076; *P* = .02 for salbutamol and of 0.009 Kpa/L/s, 95% CI = −0.028 to 0.045; *P* = .98 for HBB for the resistance at 3 Hz and of 0.040 Kpa/L/s, 95% CI = 0.007 to 0.074; *P* = .01 for salbutamol and of 0.009 Kpa/L/s, 95% CI = −0.024 to 0.042; *P* = .97 for HBB for the reactance at 7 Hz). From 5 to 30 minutes after HBB administration, the heart rate accelerated (mean increase of 3.3 beats per minute, 95% CI = −6.6 to 13.1; *P* = .92 for salbutamol, and of 13.0 beats per minute, 95% CI = 3.6 to 22.4; *P* = .002 for HBB at 30 minutes) and the gut sounds decreased (mean reduction of 1.3, 95% CI = −0.1 to 2.8; *P* = .09 for salbutamol and of 2.8 for the gastrointestinal auscultation score, 95% CI = 1.4 to 4.3; *P* < .0001 for HBB at 30 minutes).

**Conclusions and Clinical Importance:**

Both drugs have a similar bronchodilator potency but with a longer duration for salbutamol. Gastrointestinal and cardiovascular effects were noted only with HBB, suggesting the preferential use of salbutamol to relieve bronchoconstriction in horses with asthma.

**Abbreviations**
HR: heart rate
HBB: hyoscine butylbromide
R: resistance
SA: severe asthma
X: reactance

## 1 INTRODUCTION

Severe asthma (SA) in horses is a common and incurable disease affecting approximately 14% of the equine population in the northern hemisphere.^1^ It is characterized by airway obstruction because of inflammation, bronchoconstriction, mucus accumulation, and structural changes in the airways.^2^, ^3^ The cornerstone treatments for equine asthma are environmental and dietary changes implemented to avoid exposure to offending inhaled antigens. When these changes are not possible or are insufficient to control airway obstruction, or when rapid relief is required, administration of corticosteroids, bronchodilators, or both is indicated.^4^ Bronchoconstriction is the main cause of airflow obstruction in asthma, as demonstrated by the rapid decrease of pulmonary resistance after the administration of bronchodilators.^5^, ^6^, ^7^ The bronchial tone is regulated by the cholinergic parasympathetic system, the adrenergic sympathetic system, and the nonadrenergic and noncholinergic system.^8^, ^9^ These neural pathways are modulated by bronchodilators commonly used in horses with asthma, including β2‐adrenergic agonists, such as inhaled salbutamol,^10^ and anticholinergic drugs, such as the muscarinic receptor antagonists atropine and hyoscine butylbromide (HBB).^11^ Although atropine was formerly considered to be the most potent bronchodilator in horses with asthma, HBB has been the preferred anticholinergic in recent years because of its shorter half‐life, which might minimize its adverse effects while maintaining equivalent efficacy.^12^ Furthermore, HBB is approved in North America and Europe (by the name of N‐butylscopolammonium bromide) to control intestinal pain associated with uncomplicated colic in horses. Nevertheless, HBB results in tachycardia and is contraindicated in horses with unstable cardiovascular status or with primary cardiovascular disease, and it can also decrease intestinal motility.^13^, ^14^ As tachycardia, right ventricular dysfunction, and pulmonary hypertension can occur during exacerbation of SA,^15^ affected horses might be more susceptible to the cardiovascular effects of this drug. Conversely, inhaled salbutamol has a favorable safety profile in SA.^10^

The primary objective of this study was to evaluate the bronchodilation duration and potency of these 2 drugs in SA, and secondarily, to compare the occurrence of adverse effects. The hypothesis was that HBB is more potent than salbutamol in relieving bronchospasm but produces more adverse effects, especially tachycardia.

## 2 MATERIALS AND METHODS

### 2.1 Horses

Using a randomized, blinded, crossover design, 8 horses with SA from a research herd (3 geldings and 5 mares, aged 17 ± 2.7 years [mean ± SE of the mean], and weighing 588 ± 85.7 kg) were studied. Horses had a history of labored breathing, airway obstruction, and lower airway inflammation when stabled and fed hay. A power analysis, based on previously acquired lung function data measured by oscillometry,^16^ determined that a sample size of 8 horses per group would be sufficient to detect a 20% difference in airway resistance with a power of 80% and a significance level (alpha) of 5%. All procedures were approved by the Animal Care Committee of the Faculty of Veterinary Medicine of the Université de Montréal (Protocol # 21‐Rech‐2155).

Before the study, concomitant diseases were excluded in all horses by a clinical examination and a CBC analysis. To induce asthma exacerbation (labored breathing and airway obstruction documented by oscillometry), susceptible horses were stabled and fed hay for 3 weeks before the study, with daily access to a paddock. The attitude, appetite, and presence of severe respiratory distress were evaluated daily. Endpoints requiring additional treatments included tachycardia greater than 60 bpm, respiratory distress with a score of 7 or 8/8,^17^ fever, lameness, or signs of colic.

### 2.2 Study design

Using a crossover design, 4 horses were first administered 1000 μg of salbutamol (Ventolin, GSK, Mississauga Road Mississauga, Ontario, Canada) using an inhalation device (Equine AeroHippus, Trudell Medical International, London, Ontario, Canada), whereas the 4 other horses received 150 mg of HBB intravenously (Buscopan, Boehringer Ingelheim, South Service Road, Burlington, Ontario, Canada). Each drug was administered once in the morning, and the treatments were reversed after a 72‐hour withdrawal period. Group allocation was based on the ranking clinical severity of the respiratory difficulty, and a coin toss allocated the treatment order. Lung function and abdominal auscultation of gastrointestinal sounds (score 0‐3)^18^ were blindly evaluated before and 5, 10, 15, 30, 60, 90, 120, and 180 minutes after treatment. The monitoring of the heart rate was not blinded, as we judged that the occurrence of tachycardia would likely alert the blinded evaluator to the higher possibility that the horse could be receiving HBB, and, therefore, influence the assessment of the other parameters.

### 2.3 Lung function measurement

The lung function was measured using oscillometry (Equine IOS MasterScreen, Jaeger, Würzburg, Germany) as previously described.^19^ In brief, acclimatized horses were placed in a stock without sedation. An airtight facemask connected to the oscillometry device was placed, and the head was maintained in a physiological position. Oscillometry assesses the impedance of the respiratory system, which encompasses the factors that counteract the transmission of sound waves. It is measured through 2 parameters: pulmonary resistance (R) and reactance (X). Resistance reflects the opposition encountered by the airflow, primarily attributed to the central airways. Reactance assesses the elastic and inertial properties of the lungs.^20^ The Equine IOS uses a speaker that produces short‐pressure pulses sent into the airways and superimposed on the animal's normal breathing. The whole‐breath resistance and the reactance values of the respiratory system were calculated using flow and pressure signals provided by a pneumotachograph and differential pressure transducers.^19^ Values were recorded with LabManager (version 4.53, Jaeger, Würzburg, Germany) and then analyzed following Fast‐Fourier transformation (FAMOS IMC, Meβsysteme, Berlin, Germany). At least 3 measurements of 30 seconds each were recorded before and at 5, 10, 15, 30, 60, 90, 120 and 180 minutes after treatment administration. The data at 3 and 7 Hz were examined. Higher frequency impulses have a shorter travel distance and primarily indicate central and upper airway changes. Lower frequency impulses penetrate deeper into the lungs, enabling the detection of peripheral airway dysfunction observed in conditions like asthma.^21^, ^22^ The ratio of the resistance at 3 and 7 Hz was used to assess the frequency‐dependence of the airway obstruction, as more severe lung dysfunction in smaller airways is expected during exacerbation. Only values with coherence >0.85 at 3 Hz and >0.9 at 7 Hz were considered for analysis.

### 2.4 Statistical analysis

Data were analyzed using Prism software (GraphPad Prism 10.0.1; San Diego, CA, USA). The distribution of the data was visually inspected for normality. Two‐way ANOVA, or a mixed‐effects model when some data points were missing, were used to compare the effects of both drugs on the lung function, heart rate, and gastrointestinal auscultation score, with the treatment group and time as independent variables. The duration of action was evaluated with Dunnett's multiple comparison tests. The level of statistical significance was set at 0.05.

## 3 RESULTS

The datasets generated in the current study are available in a Supplementary file.

### 3.1 Clinical signs

One horse was excluded from the study as it did not exhibit clinical signs of airway obstruction and its lung function remained normal despite exposure to the environmental challenge. Additionally, 1 mare experienced severe airway obstruction during the first treatment period, necessitating additional treatments according to the study endpoints. Data from this mare were excluded from the analysis to prevent interference with the study results. All included horses tolerated both drugs well.

### 3.2 Lung function

There were no differences in the pulmonary resistance and reactance values between the baseline data of the 2 periods of this crossover study.

#### 3.2.1 Pulmonary resistance

**FIGURE 1:**
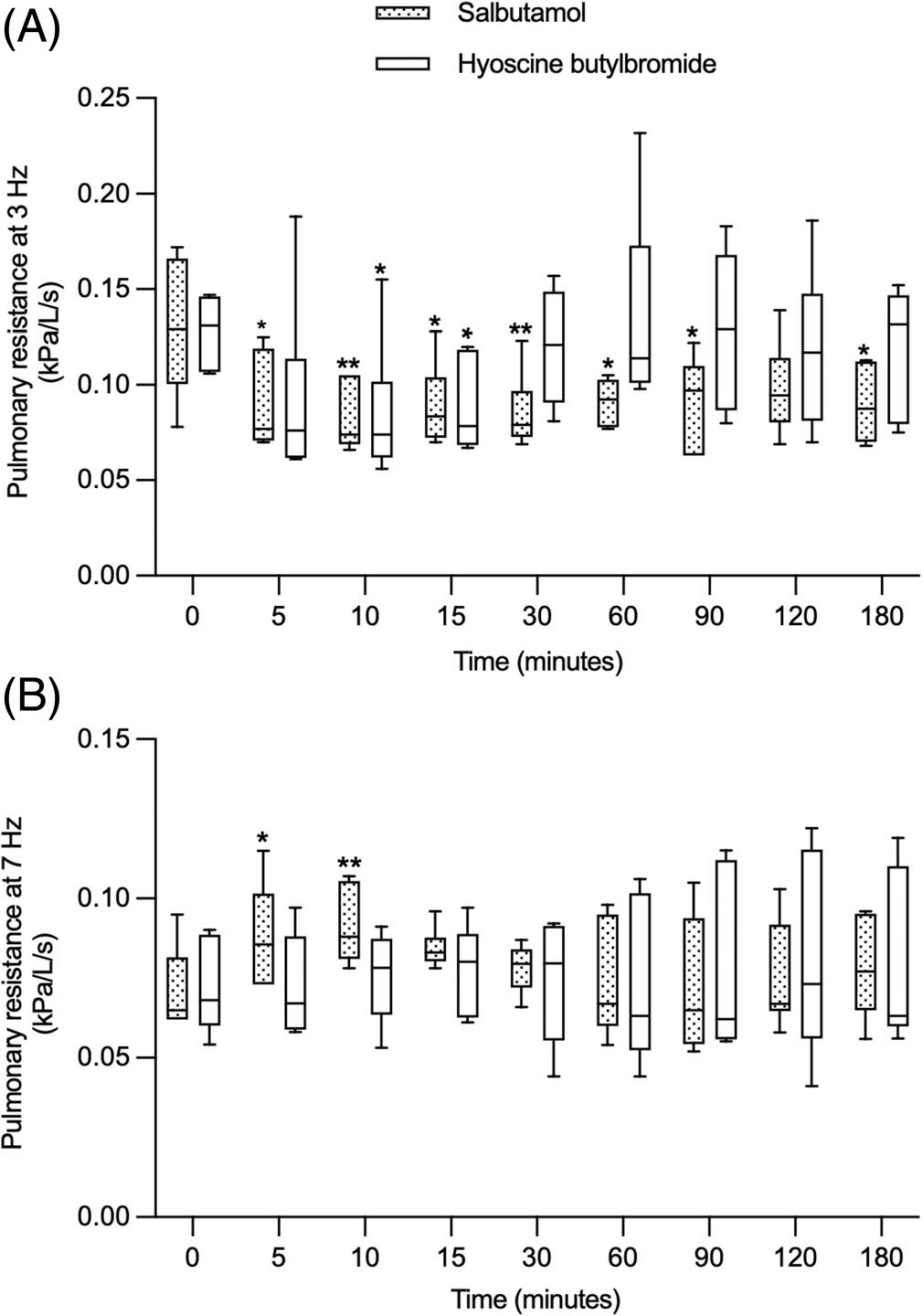
Pulmonary resistance at 3 Hz (A) and 7 Hz (B) before (0) and for 180 minutes after the administration of salbutamol and HBB (n = 6 in each group). The boxes extend from the 25th to 75th percentiles with a horizontal line at the median, and the whiskers represent the 5 and 95% percentiles. *P < .05, **P < .01, and ***P < .001 when compared from baseline within a group.

**FIGURE 2:**
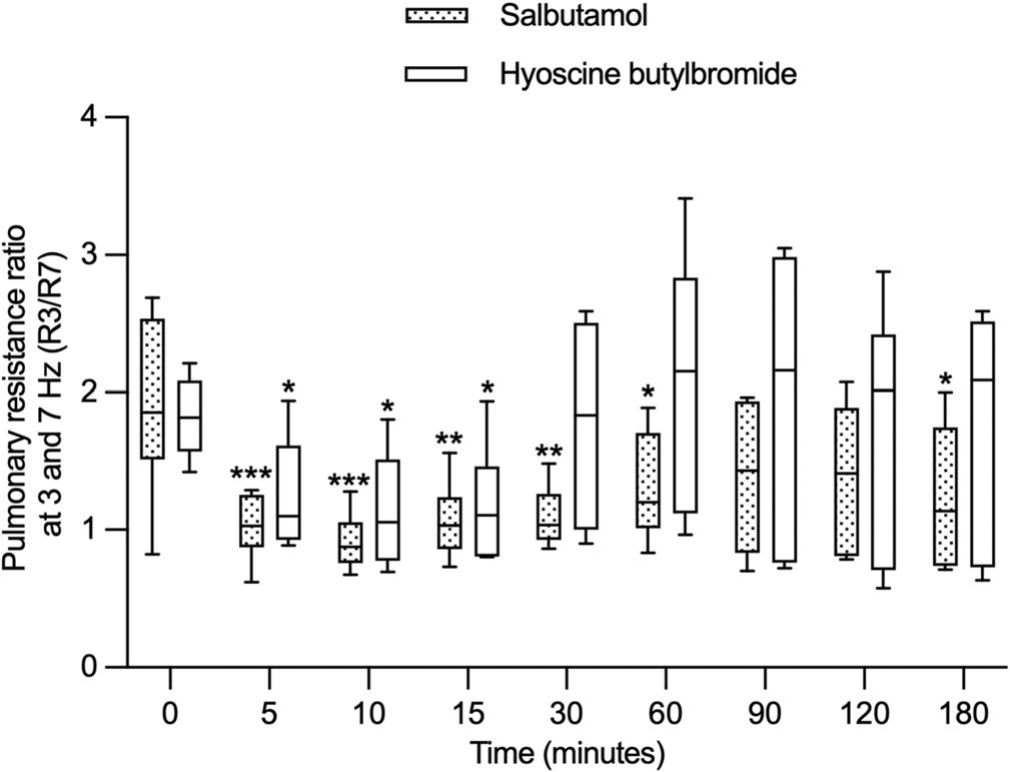
Ratio of the pulmonary resistance at 3 and 7 Hz (R3/R7) before (0) and for 180 minutes after the administration of salbutamol and HBB (n = 6 in each group). The boxes extend from the 25th to 75th percentiles with a horizontal line at the median, and the whiskers represent the 5 and 95% percentiles. *P < .05, **P < .01, and ***P < .001 when compared from baseline within a group.

At the lower frequency of 3 Hz, the pulmonary resistance was significantly different overtime after the administration of the bronchodilators (time effect, *P* < .001). It improved from 5 to 180 minutes (not significant at 120 minutes) after salbutamol inhalation and at 10 and 15 minutes after HBB administration (mean improvement at 180 minutes of 0.040 Kpa/L/s, 95% CI = 0.004 to 0.076; *P* = .02 for salbutamol and of 0.009 Kpa/L/s, 95% CI = −0.028 to 0.045; *P* = .98 for HBB). At 7 Hz, the bronchodilators behaved differently over time as there was an interaction between the treatment group and time of treatment (*P* = .04). With salbutamol, the pulmonary resistance at 7 Hz significantly increased at 5 and 10 minutes compared to baseline (Figure 1B) whereas it remained unchanged with HBB (mean increase at 10 minutes of 0.020 Kpa/L/s, 95% CI = 0.006 to 0.035; *P* = .002 for salbutamol and of 0.004 Kpa/L/s, 95% CI = −0.011 to 0.018; *P* = .97 for HBB). The R3/R7 ratio, a variable that deteriorates with small airway diseases such as asthma, significantly improved over time in both groups (time effect, *P* < .0001). It improved at 5, 10, 15, 30, 60, and 180 minutes with salbutamol and from 5 to 15 minutes with HBB (mean improvement at 180 minutes of 0.676, 95% CI = 0.099 to 1.252; *P* = .02 for salbutamol and of 0.047, 95% CI = −0.529 to 0.623; *P* > .99 for HBB; Figure 2).

#### 3.2.2 Pulmonary reactance

**FIGURE 3:**
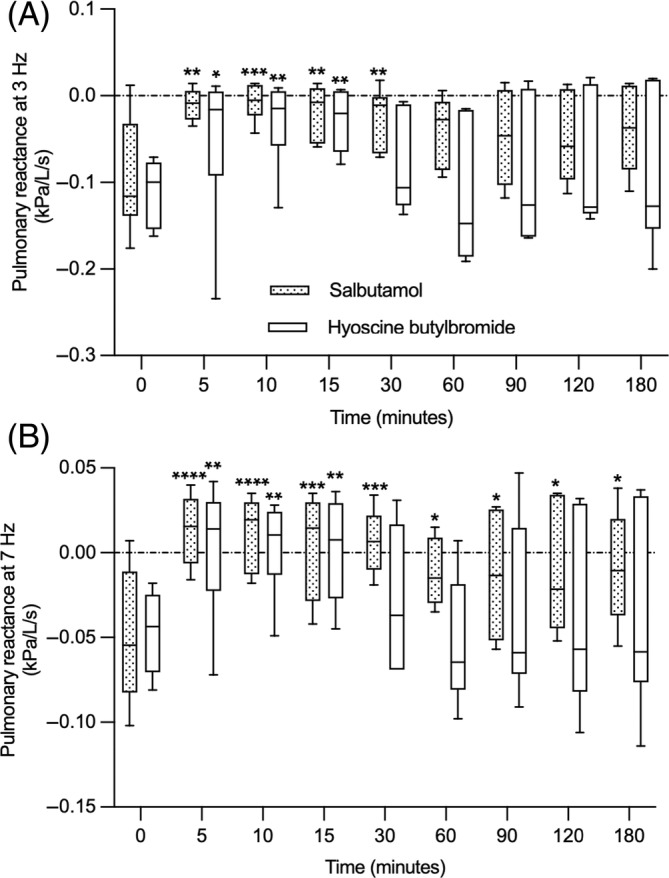
Pulmonary reactance at 3 Hz (A) and 7 Hz (B) before (0) and for 180 minutes after the administration of salbutamol and HBB (n = 6 in each group). The boxes extend from the 25th to 75th percentiles with a horizontal line at the median, and the whiskers represent the 5 and 95% percentiles. *P < .05, **P < .01, and ***P < .001 when compared from baseline within a group.

At the lower frequency of 3 Hz, the reactance, a measure of lung elasticity, improved over time in both treatment groups (time effect, *P* < .0001). It increased from 5 to 30 minutes with salbutamol and from 5 to 15 minutes (Figure 3A) with HBB (mean increase at 30 minutes of 0.07 Kpa/L/s, 95% CI = 0.013 to 0.127; *P* = .01 for salbutamol and of 0.029 Kpa/L/s, 95% CI = −0.028 to 0.086; *P* = .63 for HBB). Similarly, the reactance improved over time at the impulse frequency of 7 Hz (time effect, *P* < .0001). It increased starting 5 minutes after the administration of both bronchodilators and lasted 180 minutes with salbutamol and 15 minutes with HBB (Figure 3B; mean improvement at 180 minutes of 0.040 Kpa/L/s, 95% CI = 0.007 to 0.074; *P* = .01 for salbutamol and of 0.009 Kpa/L/s, 95% CI = −0.024 to 0.042; *P* = .97 for HBB). Of note, the same 2 horses had improved lung function (R3/R7, R3, X3, and X7) lasting 180 minutes with both drugs.

### 3.3 Adverse effects

There were no differences in the heart rate values and gastrointestinal auscultation scores between the baseline data of the 2 periods of this crossover study.

**FIGURE 4:**
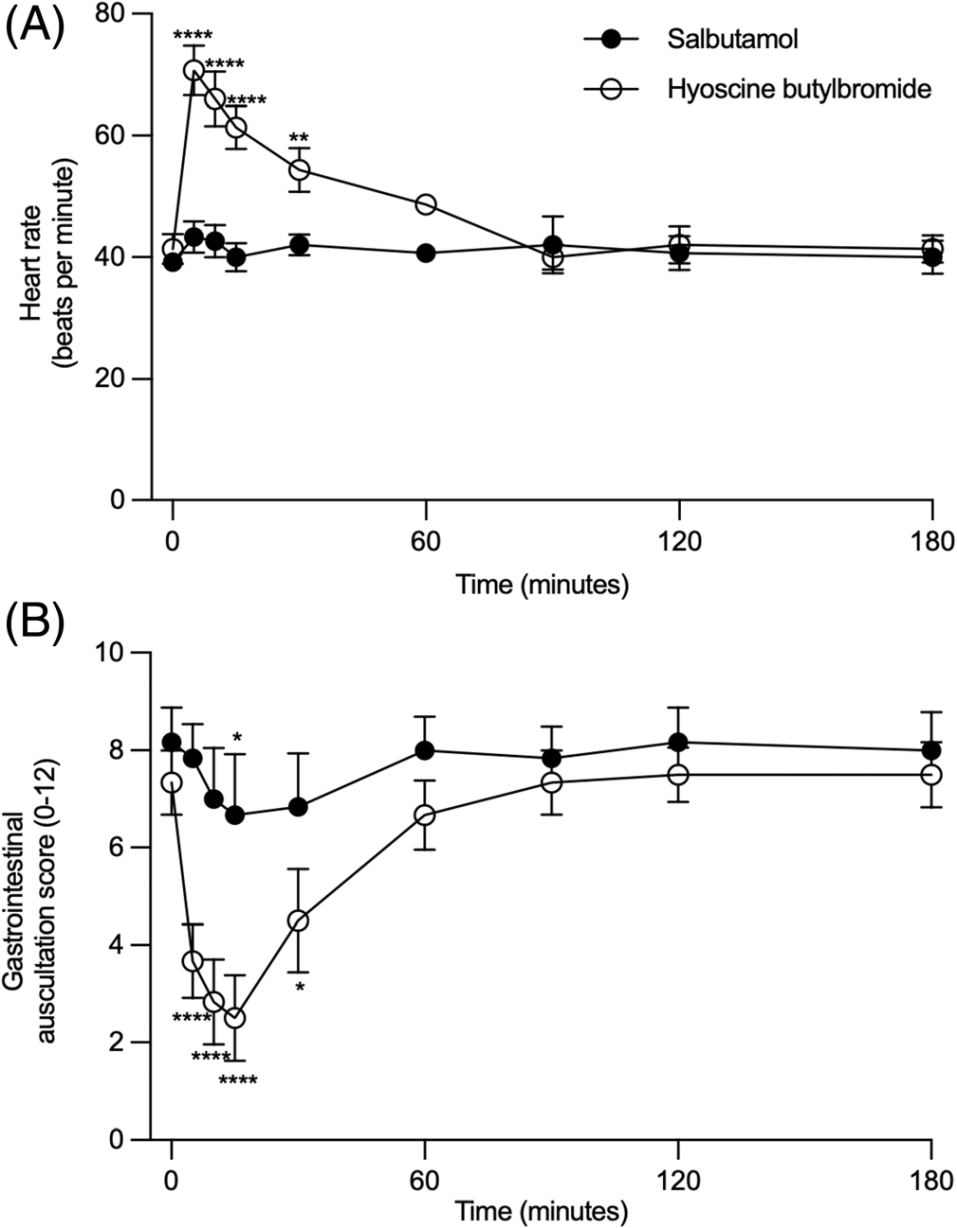
Heart rate (A) and gastrointestinal auscultation score (B; means ± SEM) before (0) and for 180 minutes after the administration of salbutamol and HBB (n = 6 in each group). *P < .05, **P < .01, and ***P < .001 when compared from baseline within a group.

There were significant effects of the treatment group (*P* = .008), time (*P* < .0001), and an interaction between the treatment group and time (*P* < .0001) for the heart rate as it only increased after the administration of HBB, for a duration of 30 minutes (mean increase at 30 minutes of 3.3 bpm, 95% CI = −6.6 to 13.1; *P* = .92 for salbutamol and of 13.0 bpm, 95% CI = 3.6 to 22.4; *P* = .002 for HBB). There were significant effects of the treatment group (*P* = .02), time (*P* < .0001), and an interaction between the treatment group and time (*P* < .0001) for the gastrointestinal auscultation score as it decreased after the administration of HBB for a duration of 30 minutes (mean reduction of the gastrointestinal auscultation score at 30 minutes of 1.3, 95% CI = −0.1 to 2.8; *P* = .09 for salbutamol and of 2.8, 95% CI = 1.4 to 4.3; *P* < .0001 for HBB). The gastrointestinal auscultation score was also significantly decreased only at the 15 minutes time point after the administration of salbutamol (Figure 4).

## 4 DISCUSSION

Bronchodilators are commonly used for the rapid relief of airway obstruction in horses, but there is limited information concerning the comparative efficacy and tolerability of these drugs. Our results indicated that salbutamol and HBB rapidly alleviate airway obstruction in horses with SA. However, although having a similar potency, bronchodilation lasted longer with salbutamol (up to 3 hours vs 1 hour for HBB) without the adverse effects observed with HBB.

Using the inhalation route of administration, the effects of salbutamol are predominantly observed in the respiratory tract, inducing smooth muscle relaxation via epinephrine.^23^ This targeted action in the lungs is a relevant advantage, as it minimizes systemic adverse events and procures the therapeutic effect where it is required. Although salbutamol does enter systemic circulation, it is rapidly metabolized,^24^, ^25^, ^26^ which can be a substantial consideration in equine competitions governed by drug use regulations. The intravenous route of administration of HBB could have contributed to its shorter duration of effect in the current study, as it directly enters the bloodstream and could be metabolized more rapidly. However, it is difficult to distinguish the role of the administration route from the distinct mechanisms of action of these molecules.

The duration of the bronchodilator effect of salbutamol in the present study (>180 minutes) is concordant with some reports in horses^10^ and in humans.^23^, ^27^ However, at lower doses (125 μg^28^ and 540 μg^29^), the bronchodilation observed in SA is of shorter duration (30‐60 minutes). Although the administration of 720 μg did not enhance bronchodilatation or increase the duration of action compared to 360 μg,^10^ we opted for the maximum dosage recommended in the current guidelines for treating asthma (2 μg/kg),^30^ because of the important variability in efficacy among different studies and the severity of airway obstruction encountered in our horses. However, we cannot exclude that a lower dose would have been equally effective. The variable duration of the bronchodilation of salbutamol reported in the different studies might be because of differences in drug formulation and the methods of administration (nebulizers or metered doses inhalers).^28^, ^29^ Also, in the current study, lung function in horses was assessed using oscillometry instead of standard mechanics as in other studies. This technique is considered more sensitive,^22^ which could explain the detection of a longer effect compared to previous reports. With oscillometry, resistance at lower frequencies evaluate the peripheral airways, whereas higher frequencies assess the obstruction occurring in central airways, the mask, and the connecting device. The site of drug deposition, the density of β2‐adrenergic receptors, and the anatomic location of bronchospasm could all have contributed to the variable duration of bronchodilation with salbutamol at the 2 different frequencies studied, with a longer duration observed with the resistance at 3 Hz and reactance at 7 Hz. However, as the duration of bronchodilation with HBB is similar to earlier reports using standard lung mechanics,^11^, ^12^ factors other than the methods of lung function measurements could have contributed to the variable results reported for salbutamol.

The paradoxical response observed at 7 Hz, characterized by a transient exacerbation in resistance over 10 minutes (not observed at 3 Hz) and an improvement of reactance lasting up to 3 hours following salbutamol administration, has not been described in other equine studies.^28^, ^29^ This effect could potentially be attributed to the increase sensitivity of oscillometry compared to conventional lung mechanics employed in earlier studies,^22^ and could be related to the induction of a contraction, instead of a relaxation, of the equine bronchi by the (S)‐enantiomers present in the racemic mixture of salbutamol, a finding shown in vitro but not reported in vivo so far in horses.^28^ In humans and animal models, adverse effects related to the S‐enantiomer, such as airway hyperresponsiveness and allergen‐induced airway edema, have been described.^31^, ^32^ Furthermore, bronchoconstriction observed with salbutamol in infants and preterm children is associated with the acidic and hypo‐osmolar properties of the solutions nebulized.^33^, ^34^ Paradoxical bronchospasm following the use of β2‐agonists by MDI occurs in humans, with various theories proposed to explain this phenomenon.^35^, ^36^

Maximal bronchodilation persisted for 180 minutes in 2 horses with both drugs, whereas it was of a shorter duration for the 4 other horses. Mucus accumulation, cough, and bronchospasm all limit penetration of aerosol medication in horses with SA. The unpredictable amount of inhaled product reaching the lower airways could have contributed to the variable duration of bronchodilation with inhaled salbutamol. The variable duration of action of salbutamol and HBB among horses might also be attributed to the varying implication of the sympathetic, parasympathetic, and nonadrenergic noncholinergic systems between individuals. These findings indicate variability in individual responses that should be considered for effective therapy in the field.

The increased heart rate with HBB observed in our study is consistent with previous reports.^12^, ^14^ The tachycardia associated with anticholinergic agents and nonselective β‐adrenergic agonists could be detrimental in horses with SA in exacerbation as pulmonary hypertension associated with functional and structural changes of the right ventricle can be present.^15^ In the HBB group, a decrease in intestinal borborygmi lasting 30 minutes was observed, which agrees with the decrease in duodenal motility^14^ and cecal motility^37^ lasting up to 20 minutes in previous reports. In the salbutamol group, the significant decrease in intestinal borborygmi only at 15 minutes might be related to the stress experienced by the horses when placed in the stock and at the onset of the manipulations. After the initial 15 to 30 minutes, the horses were sent back to their stalls until the subsequent measurements of pulmonary function and cardiac and digestive auscultation. Tachyphylaxis is another adverse effect that has been described with β2‐adrenergic in horses and humans, but only after a chronic (weeks) administration.^31^, ^38^ It is important to note that the use of HBB and salbutamol as bronchodilators for equine asthma represents an off‐label usage of these drugs. Additionally, the administration of salbutamol via the inhaled route might not always be feasible in emergency situations because of the need for specific delivery devices, which might not be readily available to veterinarians or horse owners.

One of the limitations of this study was the use of the same drug dose for all horses despite their variable weights. This approach aimed to simulate real‐world practical considerations in the field, where weighing horses is not often feasible, and doses can only be adjusted based on the amount of drug in each actuation for salbutamol using MDI. Another limitation was the relatively small sample size after excluding 2 horses, which could have resulted in a lack of statistical power to detect some group or time differences. Based on the data from the current study, 9 to 11 horses would have been required to identify group differences in airway resistance at 30 and 60 minutes, respectively, should such a difference have existed.

In conclusion, the prolonged bronchodilation and the absence of adverse gastrointestinal and cardiovascular effects suggest the preferential use of salbutamol over HBB for short‐term relief of bronchoconstriction in horses with asthma.

## CONFLICT OF INTEREST DECLARATION

Authors declare no conflict of interest.

## OFF‐LABEL ANTIMICROBIAL DECLARATION

Authors declare no off‐label use of antimicrobials.

## INSTITUTIONAL ANIMAL CARE AND USE COMMITTEE (IACUC) OR OTHER APPROVAL DECLARATION

Approved by the Animal Care Committee of the Faculty of Veterinary Medicine of the University of Montreal (Protocol # 21‐Rech‐2155).

## HUMAN ETHICS APPROVAL DECLARATION

Authors declare human ethics approval was not needed for this study.

## Supporting information


**Data S1.** Supporting information.
